# Differential recognition of HIV-stimulated IL-1β and IL-18 secretion through NLR and NAIP signalling in monocyte-derived macrophages

**DOI:** 10.1371/journal.ppat.1009417

**Published:** 2021-04-16

**Authors:** Kathy Triantafilou, Christopher J. K. Ward, Magdalena Czubala, Robert G. Ferris, Emma Koppe, Curt Haffner, Vincent Piguet, Vipulkumar K. Patel, Heather Amrine-Madsen, Louise K. Modis, Seth L. Masters, Martha Triantafilou

**Affiliations:** 1 Immunology Catalyst, Immunology Network, GlaxoSmithKline, Stevenage, Hertfordshire, United Kingdom; 2 Cardiff University, Institute of Infection and Immunity, School of Medicine, University *hospital* of Wales, *Heath Park*, Cardiff, United Kingdom; 3 ViiV Healthcare, Research Triangle Park, North Carolina, United States of America; 4 Medicinal Chemistry, GlaxoSmithKline, Stevenage, Hertfordshire, United Kingdom; 5 Division of Dermatology, Department of Medicine, University of Toronto, Toronto, Canada; 6 Division of Dermatology, Women’s College Hospital, Toronto, Canada; 7 Inflammation Division, The Walter and Eliza Hall Institute, Victoria, Australia; Imperial College London, UNITED KINGDOM

## Abstract

Macrophages are important drivers of pathogenesis and progression to AIDS in HIV infection. The virus in the later phases of the infection is often predominantly macrophage-tropic and this tropism contributes to a chronic inflammatory and immune activation state that is observed in HIV patients. Pattern recognition receptors of the innate immune system are the key molecules that recognise HIV and mount the inflammatory responses in macrophages. The innate immune response against HIV-1 is potent and elicits caspase-1-dependent pro-inflammatory cytokine production of IL-1β and IL-18. Although, NLRP3 has been reported as an inflammasome sensor dictating this response little is known about the pattern recognition receptors that trigger the “priming” signal for inflammasome activation, the NLRs involved or the HIV components that trigger the response. Using a combination of siRNA knockdowns in monocyte derived macrophages (MDMs) of different TLRs and NLRs as well as chemical inhibition, it was demonstrated that HIV Vpu could trigger inflammasome activation via TLR4/NLRP3 leading to IL-1β/IL-18 secretion. The priming signal is triggered via TLR4, whereas the activation signal is triggered by direct effects on Kv1.3 channels, causing K^+^ efflux. In contrast, HIV gp41 could trigger IL-18 production via NAIP/NLRC4, independently of priming, as a one-step inflammasome activation. NAIP binds directly to the cytoplasmic tail of HIV envelope protein gp41 and represents the first non-bacterial ligand for the NAIP/NLRC4 inflammasome. These divergent pathways represent novel targets to resolve specific inflammatory pathologies associated with HIV-1 infection in macrophages.

## Author summary

It has been previously shown that inflammasome activation can be triggered during viral infection to produce the active cytokines IL-1β and IL-18. Our study represents a significant advance, as we now show that in fact there are distinct NLR inflammasome complexes and viral ligands for IL-1β secretion (Vpu) compared to IL-18 secretion (gp41) in response to HIV-1.

Most importantly, we show that the HIV envelope protein gp41 represents the first non-bacterial ligand for the assembly of the NAIP/NLRC4 inflammasome. HIV gp41 is a viroporin, and thus **our data demonstrates for the first time that the NAIP/NLRC4 inflammasome assembles for all pore-forming proteins, irrespective of whether they have a viral or bacterial origin.** This is critical for the host antiviral response and has broad implications for innate immunity in general.

## Introduction

Although CD4+ T-cells might be the primary target of Human immunodeficiency virus (HIV), cells of the myeloid lineage, such as monocytes, macrophages and mDCs are particularly relevant cell types capable of being the target as well as the reservoir for HIV-1 infection. They constitute a target for HIV, as they express both the primary receptor, CD4, as well as the chemokine co-receptor CCR5 and thus are able to be infected by R5 tropic as well as dual tropic strains of HIV. The significance of the infection of macrophages by HIV is well established in the fact that as the infection progresses, CD4+ T-cells are depleted and individuals become increasingly macrophage-tropic [1]; with macrophages becoming the main long-lived HIV reservoir [2,3]. Independently to their role in infection, an additional role for monocytes/macrophages and mDCs is emerging, where they contribute to a chronic immune activation leading to pathogenic mechanisms associated with HIV. This dysregulated chronic immune activation is the key factor in co-morbidities associated with HIV [4], such as cardiovascular disease [5–7], HIV-associated neurological disorders [8], HIV-associated dementia (HAD) [9], immune aging [10,11] as well as all-cause mortality [12,13]. This chronic inflammation is not only observed in individuals who are not receiving anti-retroviral therapy (ART), but also in individuals who show no detectable levels of HIV RNA in the absence of ART [14].

The mechanisms leading to HIV-associated chronic inflammation have not been fully elucidated, but such potent inflammatory responses are most likely induced by the innate immune system and the cells of the myeloid lineage armed with pattern recognition receptors must be pivotal in binding and recognising microbial and danger molecules triggering potent inflammatory responses.

There is accumulating evidence that HIV is able to trigger several pattern recognition receptors (PRRs), including Toll like receptors (TLRs) [15,16] as well as the inflammasomes [17], leading to secretion of pro-inflammatory cytokines, Interleukin-1β (IL-1β) and IL-18. TLRs are type 1 transmembrane proteins that recognize pathogen-associated molecular patterns, whereas inflammasomes are large cytoplasmic oligomeric structures that are formed by members of the NOD-like receptor (NLR) family [18–20]. Inflammasome activation involves a two-step process: a priming signal delivered by pattern recognition receptors (PRRs), such as TLRs, which leads to the generation of pro-IL1β and pro-IL18 (Signal 1), and a second signal (Signal 2) for the activation of caspase-1, which in turns cleaves pro-IL1β and pro-IL18 and induces the release of the active form of the pro-inflammatory cytokines. In this way, the inflammasome acts as a signalling platform for inducing caspase-1 activation, which further leads to maturation and secretion of IL-1β and IL-18 [21]. IL-1β is one of the major cytokines that modulate the host response to intracellular pathogens, including viruses [18]. IL-18 is another critical anti-viral cytokine that is triggered by the host innate immune response in order to increase the cytolytic potential of NK cells [22,23]. Although some inflammasome sensors are capable of directly detecting pathogen associated molecular patterns (PAMPs), most are indirect sensors of homeostasis altering molecular processes (HAMPs) [24]. NLRP3 for example detects a wide range of HAMPs associated with K^+^ efflux from the cell [25], while the NAIP/NLRC4 inflammasome directly detects bacterial PAMPs, such as flagellin and the type III secretion system [26].

In the case of HIV, it has been shown that HIV structural proteins can act as PAMPs for TLRs and in particular TLR2 [15] and TLR4 [27,28], whereas its ssRNA triggers TLR7/TLR8 innate immune responses [16,29–31].

In addition, it has been shown that HIV-1 infection engages the NLRP3 inflammasome complex [17,32], however the mechanisms that regulate IL-1β and/or IL-18 have remained mostly unexplored. Questions that remain are how HIV triggers inflammasome activation? What triggers Signal 1 and Signal 2 of inflammasome activation? Which TLRs and NLRs are involved? Is the mechanism of IL-1β and IL-18 secretion in response to HIV identical? Addressing these questions is of particular importance, especially since chronic raised serum levels of IL-18 have been found in HIV patients and are associated with disease progression and pathology [33]. It has been even suggested that they may contribute to virological treatment failure in HIV-1-infected patients [33]. Given the central role that monocytes/macrophages play in the chronic inflammatory state and activation of the inflammasome observed in HIV patients, this study has focused on the mechanisms of HIV-induced inflammasome activation in primary monocyte-derived macrophages (MDMs). Here we show that IL-1β induced by HIV is predominantly regulated by the NLRP3 inflammasome, while IL-18 production is predominantly regulated by the NAIP/NLRC4 inflammasome. In contrast to previous reports, the mechanism of NLRP3 activation is from the action of Vpu, triggering K^+^efflux via Kv1.3 channels. Remarkably, NLRC4 is activated after NAIP directly binds to the HIV-1 viroporin (pore forming viral protein) glycoprotein 41 (gp41).

Thus, this is the first report which shows NAIP/NLRC4 involvement in viral recognition in MDMs, which could be conserved amongst pathogen pore-forming machinery. Our results provide novel mechanistic insight into host recognition of HIV in MDMs, which are likely to be critical determinants to the chronic immune activation associated with HIV-1 disease.

## Results

### HIV infection triggers inflammasome activation and IL-1β and IL-18 secretion

**Fig 1:**
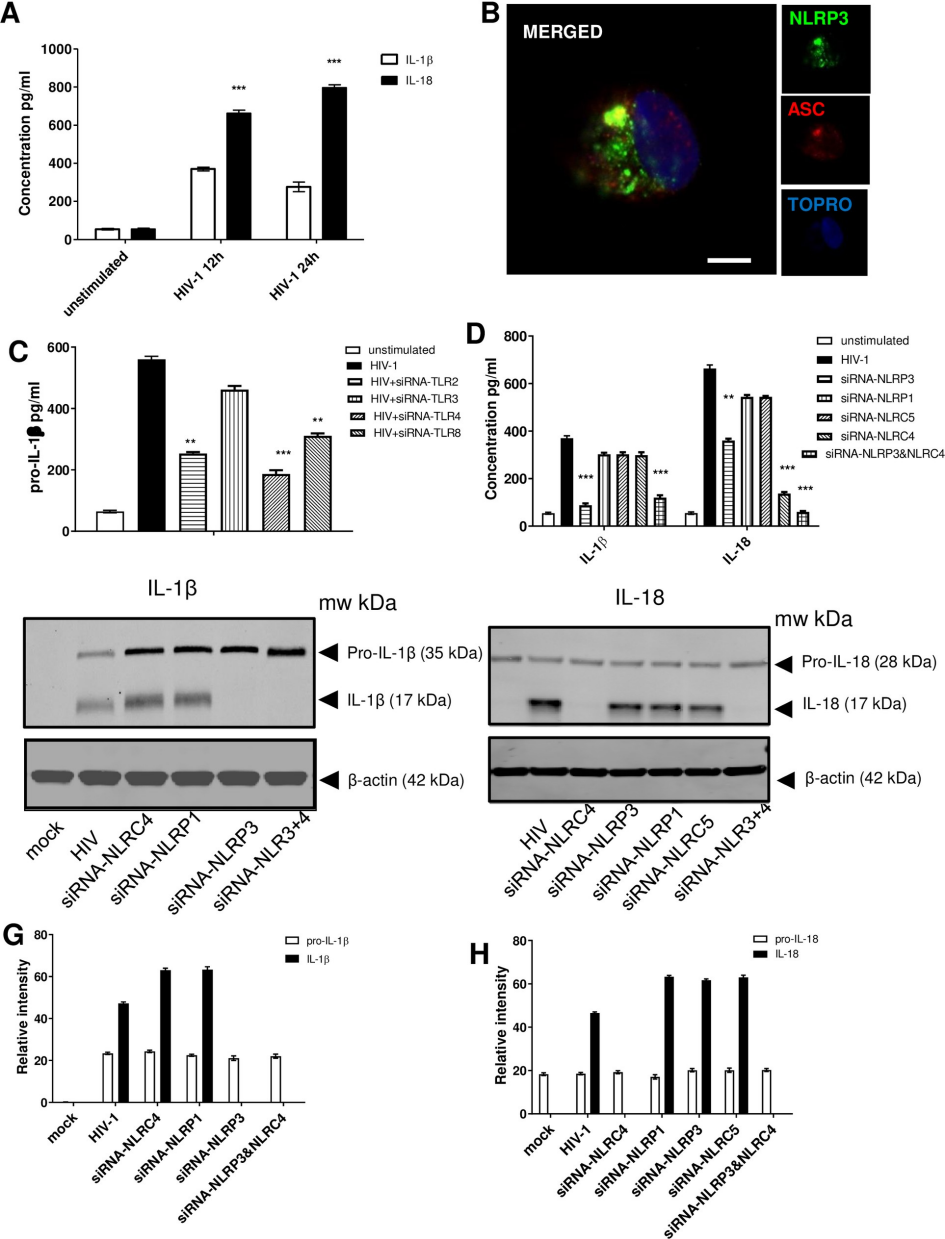
IL-1β and IL-18 secretion in HIV infection are differentially controlled. Monocyte derived macrophages (MDMs) (1 x 10^6^) were infected with HIV-1 (MOI = 1) or left untreated for 12hr and 24hr. Supernatant was collected post infection and analysed for IL-1β and IL-18 secretion using ELISA kits (R&D Systems) (A). Confocal microscopy of ASC speck formation and oligomerisation of NLRP3 in MDMs in response to HIV-1 (B). TLR2, TLR3, TLR4 or TLR8 expression in MDMs was knocked down using siRNA (80% inhibition of expression of TLRs) and infected with HIV-1 for 12 hr and subsequently determined pro-IL-1β by ELISA (C). NLRP3, NLRP1, NLRC4, or NLRC5 expression in MDMs was knocked down by siRNA (80% inhibition of expression of NLRs) and the cells were infected with HIV-1 for 12 hr and examined for IL-1β, IL-18 (D), Graphs were normalized to the level of siRNA knockdown. Furthermore NLRP3, NLRP1, NLRC4, or NLRC5 expression was knocked down by siRNA and the cells were infected with HIV-1 for 12 h and the presence of pro-IL1β, pro-IL18, and cleaved IL-1β and IL-18 was investigated via western blotting (E). Quantification of relative levels of pro-IL-1β/IL-1818 and IL-1β/Il-18 was determined by densitometry using Image Studio Lite (Licor) and normalized to internal control (β-actin) from 3 western blots (F). The data represent the mean of three independent experiments ± SD (*n* = 3 sets of MDMs) yielding consistent results. **, *p* < 0.005 and ***, *p* < 0.001 indicate statistically significant differences.

Given that IL-18 and IL-1β are elevated in the serum of HIV patients [34,35], we proceeded to model this *in vitro* by measuring IL-1β and IL-18 secretion from primary infected monocyte-derived macrophages (MDMs). We saw that HIV infection resulted in inflammasome activation and IL-1β and IL-18 secretion within 12 hr with an increase of IL-18 at 24 hr (Figs 1A and S1). Inflammasome activation was further confirmed using confocal microscopy in order to determine whether there is Apoptosis-associated speck-like protein containing a CARD (ASC)-speck formation in response to HIV. ASC speck formation has a pivotal role in inflammasome assembly and activation. It was shown that HIV induced ASC speck formation of NLRP3 and the adaptor molecule ASC in MDMs (Fig 1B).

### HIV-induced “Signal 1” of inflammasome activation

Since inflammasome activation is a two-step process, comprising of 1) a priming signal delivered by PRRs, such as TLRs, and 2) an activation signal (Signal 2) for the activation of caspase-1 which cleaves pro-IL1β and pro-IL18 and induces the release of the active form of the pro-inflammatory cytokines; we sought out to investigate which PRR(s) trigger Signal 1. We proceeded to knock down using siRNA several TLRs and subsequently infect the knockdown cells with HIV for 12h (Fig 1C). Since IL-18 has been shown to be constitutively expressed in the blood monocytes of healthy humans [36], whereas IL-1β is not constitutively expressed under homeostasis and it is induced in myeloid cells during stimulation with TLR ligands and other cytokines [37], we proceeded to assess the generation of pro-IL-1β. It was shown that knocking down TLR2, TLR4 and TLR8 inhibited the pro-IL-1β production (Fig 1C), suggesting that Signal 1 must be coming from those signalling PRRs.

In order to identify a specific inflammasome component regulating the cytokine production observed, NLRP3, NLRP1, NLRC4 and NLRC5 expression was knocked down using siRNA and cells were infected with HIV-1. As expected, IL-1β production was impaired when NLRP3 was knocked down while silencing of NLRC5, NLRC4 or NLRP1 had no effect (Fig 1D). In contrast, silencing NLRP3 only had a partial effect on IL-18 levels, silencing NLRP1 or NLRC5 had no effect, and we were surprised to see that silencing NLRC4 led to a large, significant decrease in IL-18 production in the presence of HIV-1. When both NLRP3 and NLRC4 were silenced IL-18 production was returned to baseline. This demonstrates that secretion of IL-1β and IL-18 is regulated differentially in response to HIV-1 (Fig 1D). This was further shown by western blotting, where in the knockdown cells it was shown that pro-IL-1β was induced in response to HIV and subsequently cleaved into the active form only when NLRP3 was expressed (Fig 1E and 1G); whereas pro-IL-18 was found as expected to be constitutively expressed and to be cleaved into the active form only when NLRC4 was present (Fig 1F and 1H).

### HIV viroporins trigger inflammasome activation

In order to determine which HIV component triggers inflammasome activation, we proceeded to test HIV proteins which have been previously shown to trigger inflammatory responses. MDMs were stimulated with HIV gp41, gp120 as well as Vpu in order to determine whether the proteins themselves, and not the whole virus, can trigger IL-1β/IL-18 responses (S1A and S1B Fig). Initially MDMs were stimulated with different concentrations of Vpu (S1A Fig) or gp41 (S1B Fig) in order to determine the optimum concentration to use for our stimulations. We also tested whether the transfection/vehicle reagent itself, Lyovec, was eliciting any responses (S1C Fig), which was shown to be inert. In addition, in order to determine whether the levels of HIV proteins upon infection by HIV reflected the levels of Vpu or gp41 used for our stimulations, MDMs were lysed following either HIV infection (12 hr) or 12 hr stimulation with different concentrations of Vpu/gp41 proteins and analysed by western blotting (S1D Fig). When quantified by densitometry it was shown that when 100 ng/ml of Vpu/gp41 were used, there was three times as much Vpu/gp41 protein when compared to the protein concentration resulting from HIV infection (S1D Fig). In order to use a physiologically relevant concentration of the proteins for our experiments, we decided to utilise 50 ng/ml for all our subsequent stimulations, which roughly equates to the protein levels that are found when the cells are infected with HIV. To verify this, we either infected MDMs with HIV-1 for 12 hr or stimulated MDMs with 50 ng/ml Vpu or gp41 and assessed IL-1β and IL-18 responses (S1E Fig). It was shown that at this concentration, Vpu/gp41 elicited similar responses to the virus itself.

**Fig 2:**
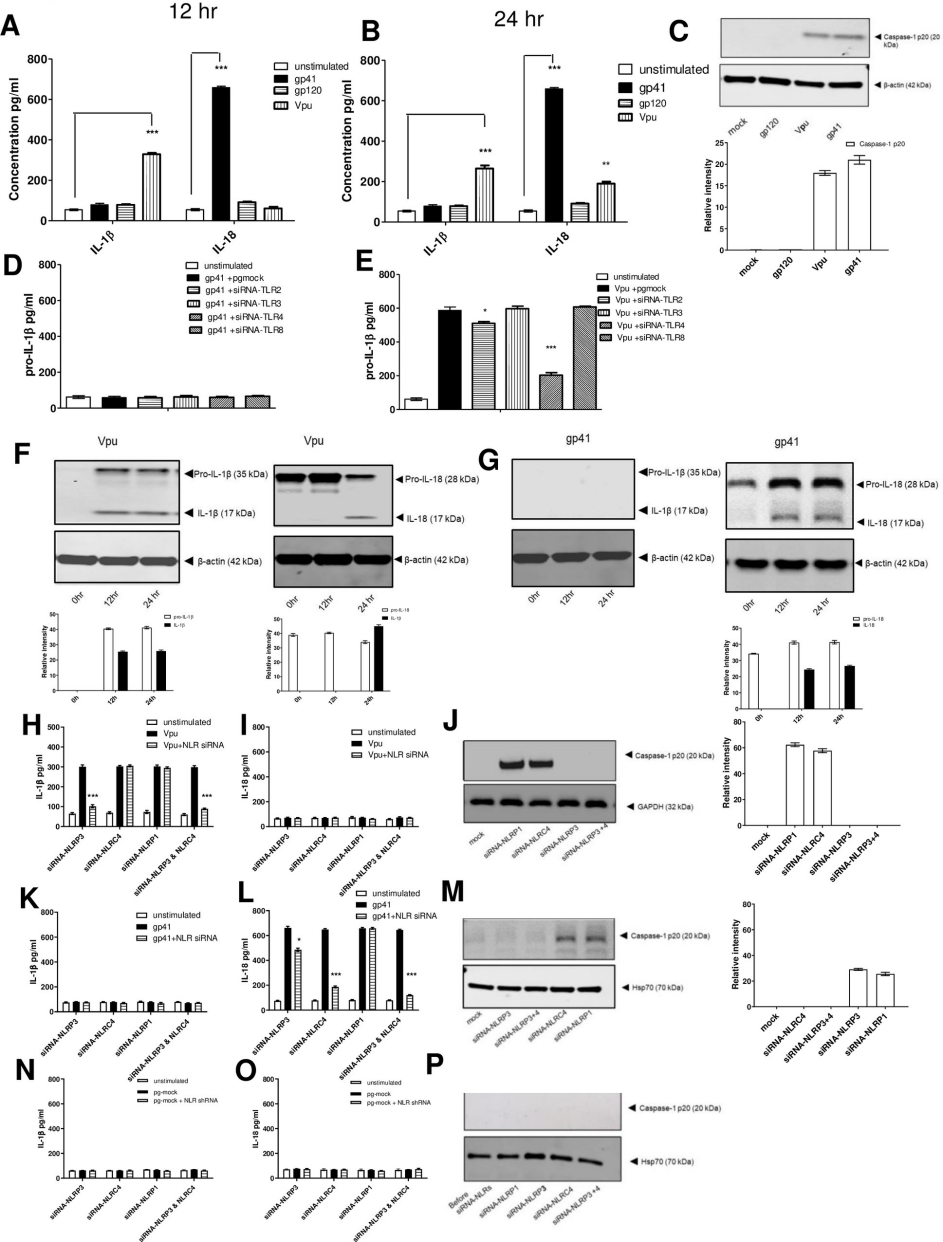
HIV Vpu and gp41 play a role in inflammasome activation. Monocyte derived macrophages (MDMs) (1 x 10^6^) were either left untreated or transfected with 2μg of pVpu plasmid expressing Vpu or pgp41 or gp120 plasmid expressing gp120. Supernatants were collected and tested for IL-1β and IL-18 secretion at 12 hr (A) and 24 hr (B). Cells extracts at 12 hr from mock or transfected cells were analysed for the presence of caspase-1 p20 by western blotting, followed by quantification using Image Studio Lite (Licor) and normalized to internal control (β-actin) from 3 western blots(C). MDMs expressing gp41 (D) or Vpu (E) monocytes were silenced for TLR2, TLR3, TLR4 and TLR8 by siRNA. Supernatant was collected at 12 hr and analysed for IL-1β and IL-18 using ELISA. Cell extracts of MDMs stimulated with either Vpu (F) or gp41 (G) were analysed for the presence of pro-IL-1β, pro-IL-18, cleaved IL-1β or cleave IL-18 by western blotting, followed by quantification using Image Studio Lite (Licor) and normalized to internal control (β-actin) from 3 western blots. MDMs expressing Vpu (H-J), gp41 (K-M) or pgmock (N-P) were silenced for NLRP3, NLRP1, NLRC4 or NLRP3 and NLRC4 by siRNA. Supernatant was collected at 12 hr and analysed for IL-1β (Η, Κ, Ν) and IL-18 (I,L,O) using ELISA. All graphs were normalized to the level of siRNA knockdown. Cells extracts were analysed for the presence of caspase-1 p20 at 12 hr by western blotting, followed by quantification using Image Studio Lite (Licor) and normalized to internal control (β-actin) from 3 western blots (J,M,P). The data represent the mean of three independent experiments ± SD (*n* = 3 sets of macrophages) yielding consistent results. **, *p* < 0.005 and ***, *p* < 0.001 indicate statistically significant differences.

Since we had established the concentration to be used for the proteins, we proceeded to stimulate MDMs with HIV viroporins, It was shown that gp120 was not able to trigger inflammasome activation on its own, Vpu was found to be able to trigger both IL-1β and IL-18 in a time-dependent manner, whereas gp41 was able to trigger only IL-18 production (Fig 2A and 2B). The inflammasome activation observed in response to Vpu and gp41 was further confirmed by the detection of the activated component of caspase-1, caspase-1 p20 (Fig 2C). Since differences in the induction kinetics of IL-1β and IL-18 secretion in response to Vpu and gp41 were observed, we proceeded to perform a time course. It was shown that Vpu was able to induce IL-1β as early as 4–8 h after stimulation, but this was rapidly diminished by 24–36 h (S2A and S2B Fig). Whereas Vpu was able to induce IL-18 secretion much later, with secretion picking at 24 h and being sustained up to 36 h (S1 Fig). As expected, gp41 was not able to trigger IL-1β production, but it was able to trigger IL-18 secretion that was sustained for 36 h (S2 Fig).

In order to determine which PRRs might provide “Signal 1” of inflammasome activation for these proteins, we proceeded to knockdown different TLRs. It was shown that none of the TLRs tested provided the “priming” signal for gp41 (Fig 2D), whereas TLR4 and to a lesser extent TLR2 provided the “priming” signal for Vpu (Fig 2E).

In order to determine whether gp41 can trigger “Signal 1” of inflammasome activation, we looked at pro-IL-1β and pro-IL-18 expression in MDMs stimulated with either gp41 or Vpu. Our results showed that pro-IL-18 was constitutively present in MDMs, however as expected pro-IL-1β was not (Fig 2F and 2G). Vpu was able to induce pro-IL-1β and also cleaved IL-1β, suggesting that it was able to trigger both signals of inflammasome activation (Fig 2F). On the other hand, gp41 did not induce pro-IL-1β, but was only able to cleave the constitutively expressed pro-IL-18 into active IL-18 (Fig 2G), suggesting a one-step inflammasome activation.

In light of the effect of Vpu and gp41 in IL-1β and IL-18 production, we investigated their effect on inflammasome activation by knocking down NLRP3, NLRC4 or NLRP1 using siRNA in cells expressing either Vpu (Fig 2H–2J) or gp41 (Fig 2K–2M). When IL-1β and IL-18 secretion as well as caspase-1 p20 expression was examined at 12 hr, the data showed a significant reduction in IL-1β secretion as well as a reduction in caspase p20 expression when NLRP3 was knocked down in cells expressing Vpu (Fig 2H–2J), while there was no significant change in neither IL-1β (Fig 2H) or caspase 1 p20 (Fig 2J) when NLRC4 or NLRP1 were knocked down (Fig 2H and 2J). Silencing NLRC4 showed a reduction of IL-18 in the presence of gp41 (Fig 2K), whereas silencing NLRP3 showed only a very small decrease (Fig 2K). When both NLRP3 and NLRC4 were silenced, IL-18 production was completely inhibited. To validate these results, we also used pharmacological inhibition. We used the small molecule MCC950, a well-known inhibitor for NLRP3 [38] to verify our findings (S2C Fig). Our data showed that pre-treatment with NLRP3 inflammasome inhibitor MCC950 inhibited IL-1β in cells stimulated with Vpu whereas it had no effect in cells stimulated with gp41 for 12 hr (S2C Fig). Since we had seen that Vpu could trigger a delayed IL-18 production we repeated our experiments with Vpu for 24 hr as well (S2D Fig). Our data showed that MCC950 inhibited IL-1β and IL-18 Vpu dependent secretion confirming that Vpu signals through NLRP3. In contrast, when stimulated with gp41 for 24 hr there was no IL-18 inhibition confirming that gp41 does not trigger IL-18 via NLRP3 (S2D Fig). In addition, we used a caspase-1 inhibitor, Ac-YVAD-cmk, which abrogated IL-1β and IL-18 secretion at 12 hours (S2C Fig) confirming that the secretion of the cytokines observed is caspase-1 dependent.

VSV-G pseudotyped HIV1 mutants lacking Env expression (HIV1Δenv) or lacking Vpu (HIV-1 ΔVpu) were also used to infect macrophages for 12 hr. The results showed a reduced IL-1β production in response to HIV-1 ΔVpu (S2E Fig) but a robust IL-18 response (S2F Fig), whereas when HIV1Δenv was used we saw as expected a decrease in IL-18 production with minimal change on IL-1β production (S2E and S2F Fig).

### HIV-induced “Signal 2” of inflammasome activation

**Fig 3:**
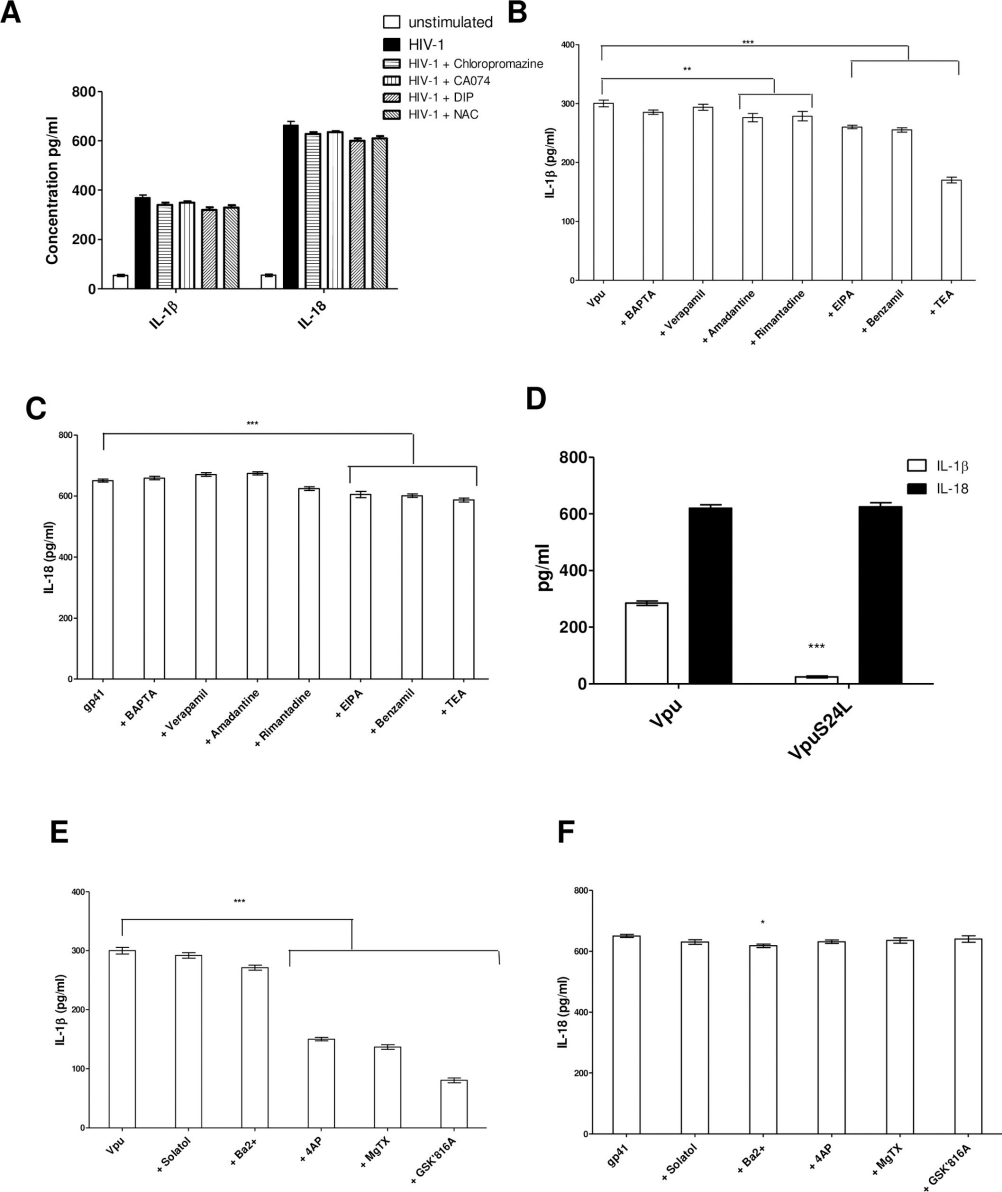
Monovalent Ion channel inhibitors reduce IL-1β dependent inflammasome activation. Monocyte derived macrophages (MDMs) (1 x 10^6^) were infected with HIV-1 (MOI = 1) for 12 hr were also cultured in the presence or absence of Cathepsin B inhibitor (CA-074) (100 μM), DPI (20μM), NAC (20mM) chloropromazine (50μg/ml). Supernatants were collected and analysed for IL-1β and IL-18 using ELISA (A). MDMs stimulated with either Vpu (50 ng/ml) (B) or gp41 (50 ng/ml) complexed with Lyovec (C)were pre-treated with and cultured in the presence of BAPTA-AM (15 μM), EIPA (25 μM), benzamil (50μM), verapamil (50μM), amantadine (6.25 μM) or rimantadine (6.25 μM) or TEA (10mM) for 12 hrs. Supernatants were collected and tested for IL-1β (B) or IL-18 (**C**)) secretion using ELISA. IL-1β secretion in response to VpuS24L (D). In addition, MDMs stimulated with Vpu (50 ng/ml) or gp41(50 ng/ml) were treated with and cultured in the presence or absence of MgTX (5nM), Ba^2+^ (300 mM), 4AP (1mM), Sotalol (100μM), GSK2332816A (350nM) for 12 hrs. Supernatants were collected and tested for IL-1β (E) or IL-18 (F) secretion using ELISA. The data presented is the mean of three independent experiments ± SD ** *p* < 0.005 and ***, *p* < 0.001 indicate statistically significant differences.

To discover how HIV infection can trigger “Signal 2” of inflammasome activation we utilised inhibitors of several published inflammasome mechanisms including: lysosome disruption, Cathepsin B inhibitor CA-074 [39,40]; production of reactive oxygen species (ROS), Diphenyliodonium (DPI) and N-acetylcysteine (NAC) [41], as well as Ca^2+^ mobilization [42], clathrin mediated endocytosis, using Chloropromazine and siRNA against clathrin [17,43]. Our results showed that none of these small molecule inhibitors had a significant effect on IL-1β and IL-18 secretion induced by HIV-1 (Figs 3A and S4).

One remaining mechanism proposed as the unifying basis for NLRP3 activation is potassium or ion efflux [25]. In order to determine whether “Signal 2” was triggered via ion channels, we proceeded to utilise different ion channel inhibitors. Permeant Ca^2+^ chelator, BAPTA-AM as well as ion channel inhibitors: 5-(*N*-ethyl-*N*-isopropyl) amiloride (EIPA) and benzamil, (blocks Na^+^/H^+^ ion channels) tetraethylammonium (TEA), (a broadly acting blocker of potassium (K^+^) channels), and verapamil, (blocks Ca^2+^ channels [44]) as well as amantadine or rimantadine (blocks the influenza M2 channel [45]) were used. MDMs stimulated with Vpu or gp41 were treated with different concentrations of each compound which did not affect cell viability (viability of cells was determined by using 0.2% trypan blue and examining cells under a microscope) (S3 Fig).

Cell-free supernatant was collected at 12hr post-stimulation and analysed for IL-1β (Fig 3B) and IL-18 (Fig 3C). TEA and benzamil were successful in inhibiting IL-1β secretion but did not inhibit IL-18 upon HIV-1 infection. There was no downregulation when verapamil, amantadine or rimantadine were used. These results suggest that Vpu is selective for K^+^ monovalent cations. Although our data demonstrated that Vpu seems to be selective for K^+^ monovalent cations, it remains unclear whether the K+ channel blocker TEA prevents IL-1β secretion by blocking Vpu and/or any other K+ channel. In order to address this possibility we generated a VpuS24L mutant. This mutant is unable to form ion channels [46]. When we infected MDMs with VpuS24L mutant, it was shown that VpuS24L failed to trigger IL-1β production (Fig 3D).

To determine the mechanism by which K^+^ efflux facilitates inflammasome activation in HIV-1 infection we looked at different types of K^+^ channel blockers (4AP, Ba^2+^, Sotalol and MgTx) due to their different blocking mechanisms and preference for different types of K^+^ channels. Sotalol blocks human ERG K^+^ channels, 4AP inhibits Kv channels, Ba^2+^ inhibits inwardly rectifying K channels (Kirs) and Margatoxin (MgTX) blocks Kv1.3.as well as the specific Kv1.3 inhibitor GSK2332816A [47] (Fig 3E and 3F). Our results showed a great reduction in IL-1β when we blocked Voltage-gated (Kv) channels especially the use of Kv1.3 inhibitor GSK2332816A (GSK’816A) with EC_50_ value of 350 nM completely abrogated IL-1β production (Fig 3E). These findings strongly suggest that HIV-1 Vpu exposure induces K^+^ through Kv1.3 channels; however, IL-18 production was not inhibited by Kv inhibitors (Fig 3F).

### Vpu colocalization to the Kv1.3 microcluster is required for IL-1β production

**Fig 4:**
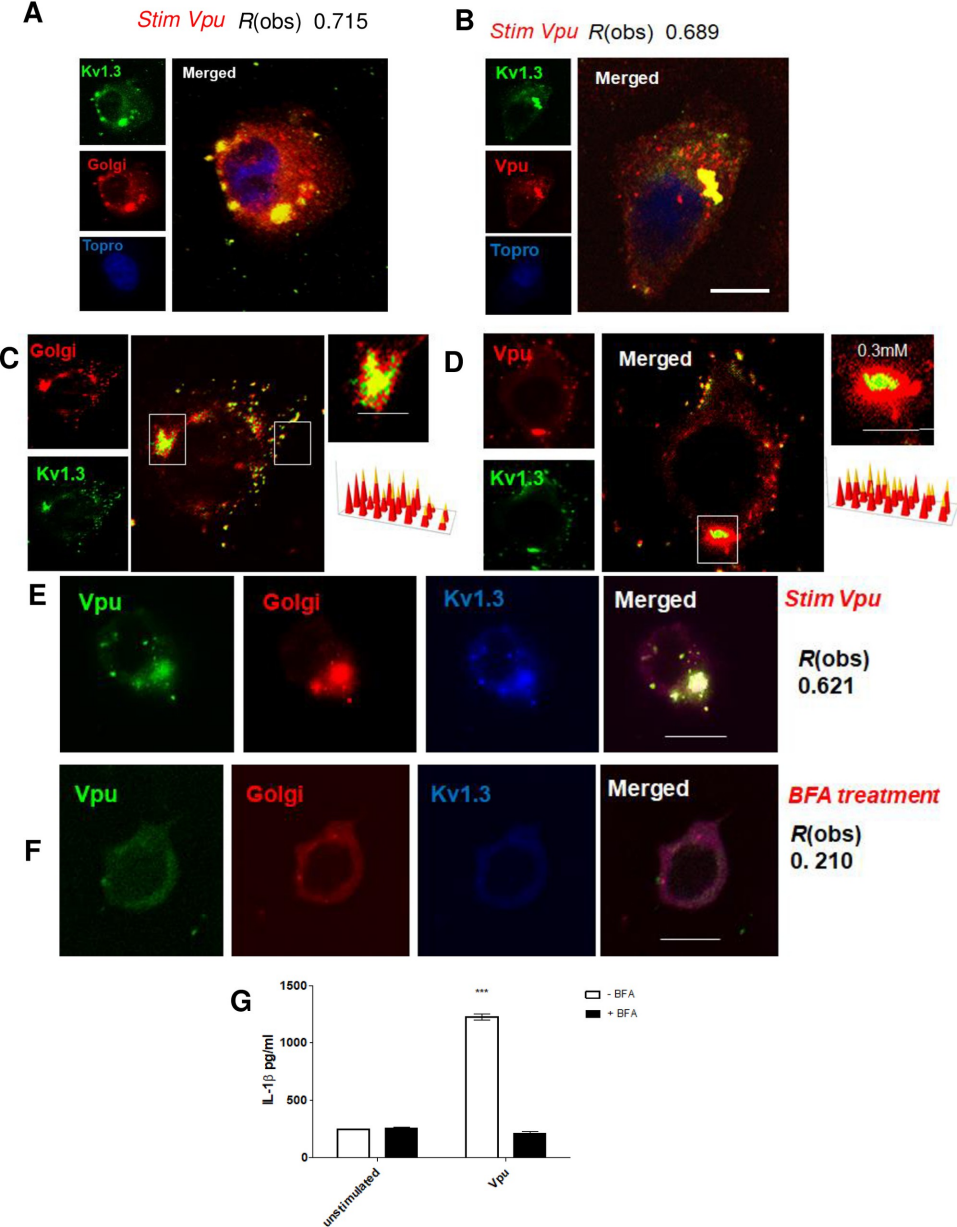
Vpu targets Kv1.3 channels to induce inflammasome activation. Monocyte derived macrophages (MDMs) were transfected with pVpu at 24 h post transfection cells were fixed and permeabilised in PBS/0.02% BSA, prior to fixation with 4% formaldehyde for 15 min. Cells were then stained with GM130 mAb to label the Golgi, a rabbit anti-Vpu antibody to label Vpu or a Kv1.3 mAb followed by the appropriate secondary conjugated to Alexa 546 (red) or Alexa 488 (green). The nucleus was stained with TOPRO-3. Cells were imaged using a Zeiss 710 confocal microscope. Bars shown are 10 μm. The data presented are at least in 20 cells over four independent experiments. The merged images show extensive colocalization between Kv1.3 and the Golgi *R*(obs) of 0.715 as well as between Vpu and Kv1.3 *R*(obs) of 0.689 respectively (A,B). The degree of colocalization was determined using ImageJ software via the Costes’ method. Data presented are representative images of n = 3 biological replicates, with at least 20 technical repeats. Findings were consistent across all replicates. SIM super resolution imaging of Kv1.3 distribution at Golgi organelles is also shown. As well as Vpu and Kv1.3 distribution in macrophages transfected with pVpu at 24 h post transfection (C,D). Quantification (right) of the relative fluorescence of the target structures where regions of interest (ROI) were selected for the target areas are shown. The distribution/position of receptors was obtained with Zeiss Zen black or ImageJ software. Cells transfected with pVpu at 24 h post transfection cells were treated with 10μg/ml Brefeldin. Golgi was stained with Anti GM130 goat antibody conjugated to Alexa 546. An anti Vpu antibody conjugated to Alexa488 was used to label the Vpu protein. Anti Kv1.3 rabbit polyclonal IgG was followed by donkey anti rabbit IgG-Alexa 633. Images of before and after treatment are depicted (E,F). Supernatants were also collected and tested for IL-1β secretion using ELISA (G). The data presented is the mean of three independent experiments ± SD ** *p* < 0.005 and ***, *p* < 0.001 indicate statistically significant differences.

To elucidate the intracellular mechanism by which Vpu triggers K^+^ efflux to activate the NLRP3 inflammasome we examined Vpu trafficking in the cell. Previous studies have shown that the Vpu protein is transported predominantly to the rough endoplasmic reticulum (RER)/ Golgi complex compartments [48], but has also been present in the recycling endosomes [49]. MDMs were transfected with pVpu for 12 hrs and permeabilised with 0.2% saponin. In order to visualise Vpu in our imaging experiments, an anti-Vpu antibody was used for labelling. The Vpu protein mainly localised at the endoplasmic reticulum and Golgi complex membranes (Fig 4A and 4B). The statistical significance of Vpu co-localisation with the Golgi was calculated by Costes’ approach, using ImageJ which returned *R*(obs) of 0.715, which are close to the theoretical maximal value suggesting that these co-localizations are highly significant (Fig 4A and 4B). It is also known that Kv1.3 channels are expressed on the plasma membrane and in the Golgi compartments in human astrocytes [50]. To determine whether this is the case in macrophages, we looked at Kv1.3 distribution. Our data showed that Kv1.3 resides in the Golgi *R*(obs) of 0.715 (Fig 4A) and moreover, it co-localises with Vpu, *R*(obs) of 0.689 (Fig 4B).

In order to obtain spatial information on Kv1.3 distribution we utilized structured illumination microscopy (SIM), which provides x and y axes resolution of ~140 nm and z axis resolution of ~250 nm. SIM imaging demonstrated that Kv1.3 channels cluster in 200–300 nm domains in the Golgi forming a microcluster (Fig 4C). Upon HIV-1 infection Vpu traffics to the Golgi and targets these microclusters (Fig 4D).

Given the Vpu co-localisation to the Kv1.3 microclusters in the Golgi we investigated the role of the Golgi apparatus in inflammasome activation by utilizing brefeldin A (BFA). The fungal metabolite BFA has been known to induce rapid and reversible disassembly of the Golgi stack into tubules and vesicles, resulting in the redistribution of Golgi-resident enzymes and accumulation of proteins in the ER in a reversible manner [51,52].

Treatment with BFA disassembled the Golgi and resulted in Vpu re-distribution (Fig 4E and 4F), which blocked inflammasome activation by HIV-1 since there was no IL-1β production in cells treated with BFA (Fig 4G). Quantification of these data using Costes’ approach indicated that BFA caused a 70% reduction in the incidence of Vpu localization to the Golgi. Taken together these observations indicate that Vpu recruitment to Kv1.3 microclusters in the Golgi is essential for triggering IL-1β dependent signalling.

### HIV-1 gp41 accumulates in lipid rafts and triggers NLRC4 via NAIP

**Fig 5:**
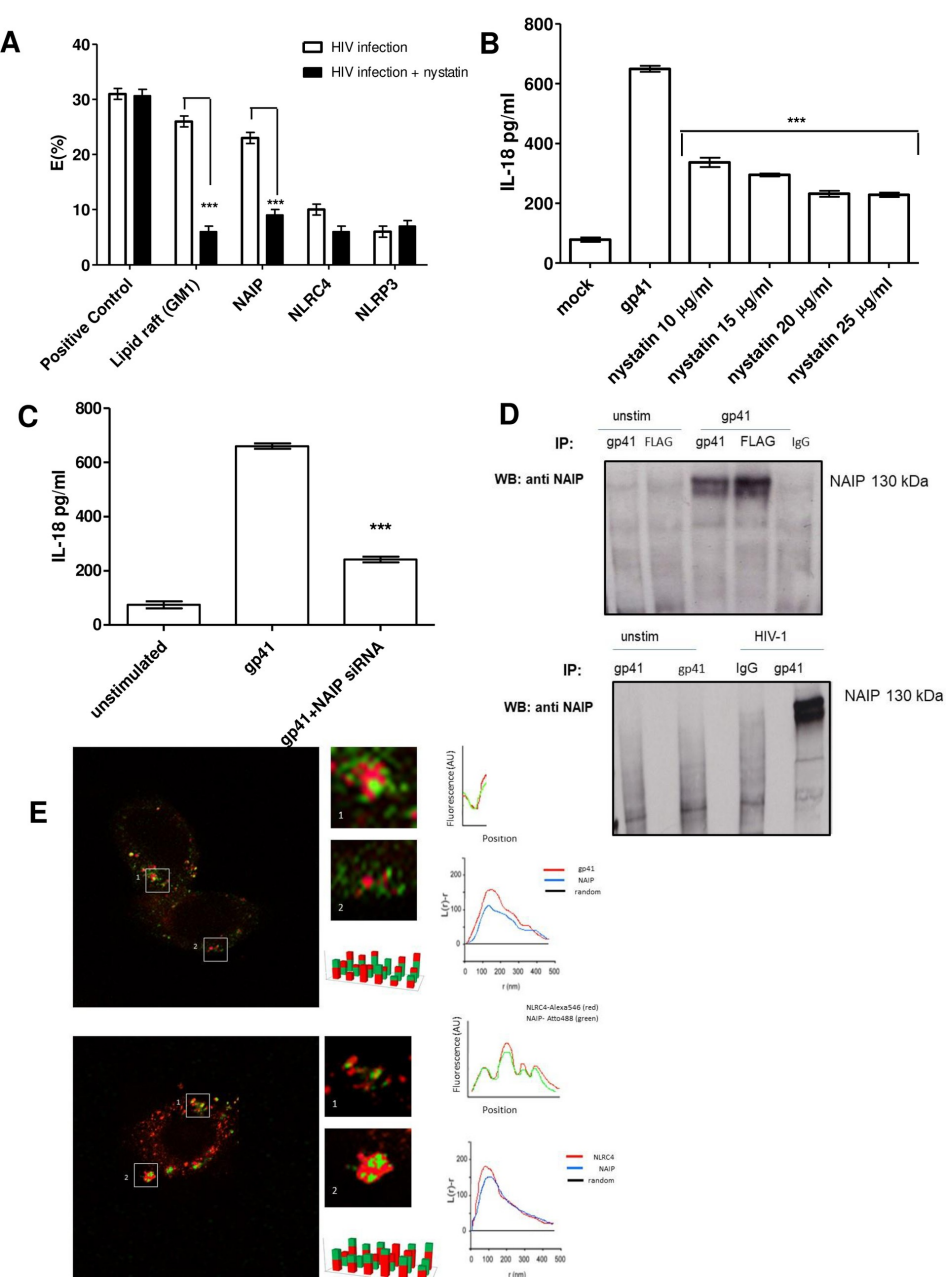
NAIP interactions with gp41. Monocyte-derived macrophages (MDMs) (1 x 10^6^) were infected with HIV-1 (MOI = 1), left untreated or pre-treated with nystatin for 12 hr. Molecular interactions between gp41 and lipid raft (GM-1 ganglioside), NAIP, NLRC4 and NLRP3 were assessed using Fluorescence resonance energy transfer (FRET) (A). MDMs stimulated with gp41 for 12 hr were treated with different concentrations of nystatin. Supernatants were also collected and tested for IL-18 secretion using ELISA (B). MDMs silenced or not for NAIP and then stimulated with gp41. Supernatant was collected and analysed for IL-18 using ELISA (C). Lysates from MDMs transfected with pgp41-FLAG (Myc-DDK tag) were subjected to IP using anti-gp41 antibody or using control IgG or using anti FLAG (D, top panel) and then analysed by western blotting using anti-NAIP antibody. Lysates from MDMs infected with HIV-1 were subjected to IP using anti-gp41 antibody or using control IgG (D, bottom panel) and then analysed by western blotting using anti-NAIP antibody. (D) The data presented is the mean of three independent experiments ± SD ** *p* < 0.005 and ***, *p* < 0.001 indicate statistically significant differences. Super-resolution images of gp41 and NAIP as well as NAIP and NLRC4 in cells infected with HIV-1 are also shown (E) Cells were labelled with anti gp41-Atto488 (green), anti NAIP-Alexa546 (red) (top panel) or anti NLRC4-Alexa546 (red) and anti NAIP–Atto488 (green) (bottom panel), fixed, imaged, and processed for dSTORM. Colocalization of receptors is presented in arbitrary units (AU) as mean fluorescence intensity of the red and green signal. Representative regions (right) of the relative fluorescence of the target structures where regions of interest (ROI) were selected for the target areas is also shown and co cluster analysis using bivariant Ripley’s L function. The distribution/position of receptors was obtained with Zeiss Zen black and ImageJ software.

To investigate how gp41 triggers a one-step inflammasome activation, we studied gp41 and NRLC4 localisation. Studies have suggested that gp41 assembly occurs within lipid-raft structures especially at the edge of the raft [53]. More specifically the cytoplasmic domain of gp41 has been shown to interact with lipid rafts [54]. Therefore GM-1 ganglioside, a raft-associated lipid, was detected using Alexa555-conjugated cholera toxin (CTX-B-Alexa555) and an anti-rabbit gp41 antibody conjugated to Alexa488 was used to label gp41. Macrophages were infected with HIV-1 and we proceeded to measure Fluorescence resonance energy transfer (FRET) between gp41 (Alexa488-anti gp41) and GM-1 ganglioside (CTX-B-Alexa555) (Fig 5A). Large dequenching was observed once the Alexa555 was photobleached (E = 26±0.9%), suggesting that gp41 concentrates in lipid rafts which contain GM-1 (Fig 5A).

In order to investigate the functional significance of lipid raft integrity we evaluated the ability of HIV to trigger inflammasome activation when lipid rafts were disrupted with nystatin, (a fungal metabolite that binds membrane cholesterol and disrupts raft integrity), or MCD, (a compound that disrupts protein association with lipid rafts) were used. It was shown that IL-18 secretion was inhibited by nystatin in a concentration-dependent manner (Fig 5B). This highlights that gp41 localises to lipid rafts, which are essential for it to activate NLRC4. At this point, it was not clear if NLRC4 directly detected gp41, or if this process requires the adaptor NAIP, which triggers NLRC4 activation in response to ligands such as flagellin and the T3SS [55,56]. To determine whether NAIP interacts with gp41, fluorescence resonance energy transfer (FRET) studies were performed. FRET can occur over 1–10 nm distances, and effectively increases the resolution of light microscopy to the molecular level. Cells were infected with HIV-1 and we proceeded to measure Fluorescence resonance energy transfer (FRET) between gp41 (Alexa488-anti gp41) and NAIP (Alexa 546 anti NAIP). The results showed strong associations between gp41 and NAIP (Fig 5A). We also analysed FRET between NAIP and gp41 as well as NAIP and NLRC4 or NLRP3. Our results showed associations between gp41 and NAIP (E = 24±1.1%) NAIP and NLRC4 (E = 20±0.7). There was no association between NAIP and NLRP3 ((E = 6±0.8%).

In order to investigate the functional significance of NAIP, its expression was knocked down using siRNA in cells stimulated with gp41. It was shown that IL-18 secretion was significantly impaired thus confirming the importance of NAIP in gp41 recognition (Fig 5C).

The interaction between gp41 and NAIP was further investigated in MDMs either infected with HIV-1 or transfected with plasmid expressing gp41. The results showed that gp41 was co-immunoprecipitated with endogenous NAIP (Fig 5D). Thus, NAIP associated with gp41 only in the presence of an HIV-1 infection.

Furthermore, we also used an alternative method to visualise the extent of NLRC4 and NAIP clustering in the presence of gp41, as well as gp41 and NAIP interactions using the super resolution microscopy method, stochastic optical reconstruction microscopy (STORM) (39). This method overcomes the resolution limit of light microscopy, allowing localisation of endogenous receptors to an accuracy of 10-30nm in fixed cells.

Cells were labelled with Fab fragments conjugated to the STORM compatible fluorophore Alexa546 (red) or Atto 488 (green), we found that in the presence of gp41, NAIP and NLRC4 are both present forming densely packed nanoclusters that resemble a speck formation. Ripley’s L-function analysis for the dSTORM images of gp41 molecules was performed for multiple cells. Here, we observed a clear co-clustering of gp41 and NAIP molecules (Fig 5F). The STORM data demonstrated that NAIP–gp41 complexes colocalize with NLRC4 providing further evidence that upon HIV-1 infection, gp41 forms clusters with NAIP and NLRC4 where it triggers inflammasome activation leading to IL-18 secretion.

### HIV gp41 CT interacts with NAIP

HIV gp41 can be divided into three major domains: the extracellular domain or ectodomain, the transmembrane domain (TM), and the cytoplasmic domain (cytoplasmic terminal tail (CT)) [57]. It has been shown that the gp41 cytoplasmic tail (CT) which extends into the cytoplasm, is important in the viral replication, infectivity, cytopathogenicity, as well as mediating association and targeting to lipid rafts [54].

**Fig 6:**
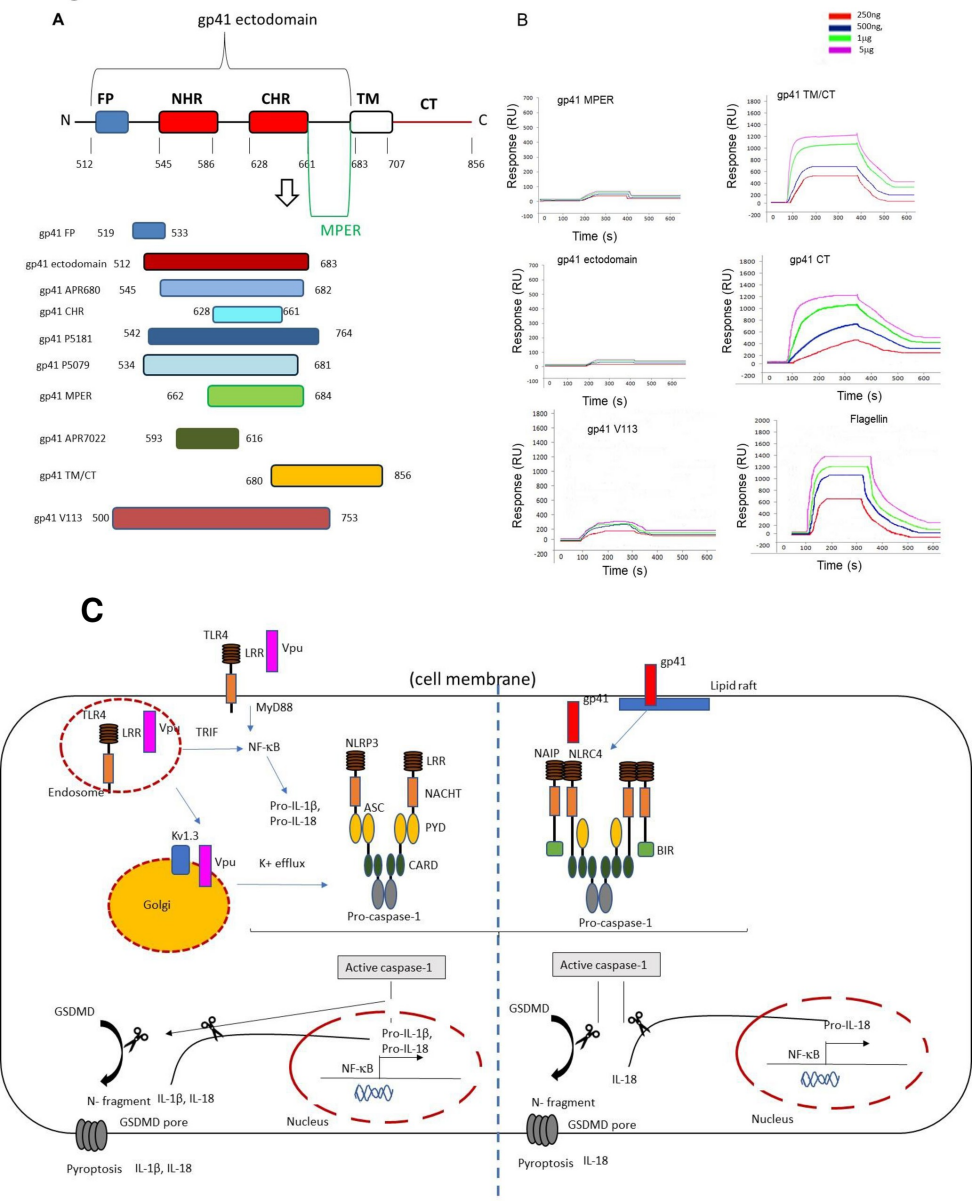
NAIP has an affinity for the CT domain of gp41. A schematic view of gp41 showing important functional regions, including the fusion peptide (FP), the transmembrane region (TM)) and cytoplasmic tail (CT). The domains are drawn approximately to scale. (A), Estimation of NAIP binding affinity to gp41 domains by the BIAcore system. NAIP protein was immobilized on the matrix of the chip and gp41 proteins were injected at flow rate 10 ml/min for 240 s. The binding ability of NAIP to each host protein was monitored and presented as a sensogram (plotted as RU versus time). For kinetic studies, gp41 proteins with increasing concentrations (500ng, 1μg, 5μg, 10 μg) were injected over the sensor chip. (B). Theoretical model of mechanisms of inflammasome activation by HIV Vpu and gp41 (C).

The ectodomain, which comprises residues 511–684, can be further broken down into a hydrophobic N-terminal domain termed the fusion peptide (FP) [58], the helical N-terminal heptad repeat (NHR), C-terminal heptad repeat (CHR) [59] and a tryptophan-rich region referred to as the membrane proximal external region (MPER) [60] that forms helical coiled-coil structures linked by a disulfide-bridged loop (Fig 6A). The primary function of the gp41 ectodomain is to drive virus–cell membrane fusion.

The TM domain amino acid sequence is highly conserved among HIV isolates and contains a conserved GXXXG helix–helix interaction motif and a highly conserved midspan arginine residue. The classical structural view of the TM is that of a single membrane-spanning a-helix [57].

To further understand the nature of gp41 and NAIP interactions and reveal gp41 domains that promote these interactions, we thus utilised peptides derived from gp41 sequence as well as gp41mutants with different sequence deletions (Fig 6B).

In this study, the binding of NAIP and gp41 was analysed by the BIAcore system. NAIP was covalently coupled through amino groups to dextran that was immobilized on the surface of the BIAcore sensor chip. Various concentrations of gp41 mutants (500ng, 1μg, 5μg, 10μg) were repeatedly injected over the immobilized protein and flagellin was used as a positive control. The sensograms were monitored as shown in Fig 6B. Our results showed that NAIP has an affinity for gp41 CT with an affinity constant (KD) 4.13x10^-10^, whereas there was no binding with the ectodomain, or the transmembrane domain (see Table 1). Thus, confirming that the region of gp41 between 707 and 856 amino acid residues is important for NAIP recognition.

**Table 1:** Binding constants of gp41 domains to NAIP. The binding affinity is expressed as KD.

Ligand Analyte NAIP	Association rate constant Ka (1/Ms)	Dissociation rate constant Kd (1/s)	Binding affinity constant (Kd/Ka) KD (M)
Flagellin	3.97 x 10^4^	9.81x10^-7^	2.47 x10^-11^
gp41 MPER			NB, no binding detected
gp 41 ectodomain			NB, no binding detected
gp41 V113	2.7 x10^1^	3.53x10^-3^	1.31x10^-4^
gp41 CT	2.39x10^4^	9.87x10^-6^	4.13x10^-10^
gp41TM/CT	4.35x10^4^	6.91x10^-5^	1.59x10^-9^

## Discussion

Anti-retroviral therapy has changed the lives of individuals infected with HIV from a death sentence to a chronic condition. As the disease phenotype changes to a chronic one, it has become apparent that the viral tropism and cellular targets also change. The R5-tropic HIV dominates in the early stages of CD4+ T-cell infection while X4-tropic virus emerges in advanced disease targeting monocytes/macrophages and mDCs [61]. Monocytes and macrophages are not only providing target and reservoir for HIV-1 infection in the later stages of the disease, but they trigger a chronic immune activation that leads to the pathogenic mechanisms associated with HIV-1 disease [62,63]. Pattern recognition receptors, such as the TLRs and the inflammasomes on monocytes/macrophages are key in triggering this inflammatory response. In particular, IL-18, which results from inflammasome activation, has been shown to be raised in the serum of individuals with advanced clinical and immunological disease and associated with virological treatment failure [33]. IL-18 seems to mediate upregulation of the HIV co-receptor CXCR4 as well as TRAIL, which is a pro-apoptotic mediator in PBMCs [33] suggesting that must be involved in CD4+ T-cell depletion and the pathogenesis of the disease.

In this study we focused on the mechanisms behind inflammasome activation in MDMs and found that HIV can trigger both IL-1β and IL-18 upon infection. Since inflammasome activation is a two-step process, we investigated which PRRs trigger “Signal 1” in response to HIV. TLR2, TLR4 and TLR8 were found to contribute to the “priming” of the MDMs, most likely via either structural components (for TLR2/TLR4) and via ssRNA for TLR8[16]. When we investigated which HIV component in particular is able to trigger the inflammasome activation it was shown that the HIV viroporin Vpu was able to trigger both IL-1β and a delayed IL-18 response, whereas gp41 was able to trigger IL-18 secretion. More interestingly, Vpu seemed to activate the NLRP3 inflammasome, whereas gp41 seemed to trigger NLRC4-induced responses.

When we investigated how the HIV proteins triggered “Signal 1” of inflammasome activation, it was found that the viroporin Vpu was able to induce pro-IL-1β production via mainly TLR4, whereas although gp41 was able to trigger inflammasome activation, it did not trigger a “priming” signal.

Previous studies have shown that distinct regulatory mechanisms can control IL-18 and IL- 1β. Zhu and Kanneganti [64] have previously demonstrated that there is a difference in the transcription of IL-1β and IL-18 in BMDMs. Therefore, it seems that IL-1β and IL-18 are differentially regulated, not only by different inflammatory signals (as in the case of Zhu and Kanneganti), but also with HIV ligands as in our study. In addition, the kinetics of activation were different. IL-1β was induced by Vpu but not sustained for long, whereas IL-18 was induced later on and sustained for longer. This is agreement with Zhu and Kanneganti and explains why there is a sustained chronic cleaved IL-18 in the serum of HIV patients.

Our results demonstrate that there is not only distinct regulatory mechanisms that control IL-1β and IL-18 production during HIV infection, but that HIV components are able to trigger different components of the innate immune sensing machinery.

HIV Vpu has the capacity to induce pro-IL-1β production and to subsequently activate caspase—cleavage resulting in secretion of IL-1β and a delayed IL-18 response via NLRP3 activation. Therefore, Vpu is able to trigger both “Signal 1” via mainly TLR4 as well as “Signal 2” of inflammasome activation. Vpu is a class IA viroporin that is mainly located at the Endoplasmic reticulum (ER) and Golgi disrupting ionic balances and is able to trigger “Signal 2” via ion flux. [65] (Fig 6C). With the use of ion channel inhibitors as well as specific K^+^ ion channel inhibitors we have demonstrated that Vpu causes K^+^ efflux possibly via membrane depolarization in the Golgi. This K^+^ efflux is mediated by opening of Kv1.3 ion channels which facilitate the maturation and release of IL-1β and a delayed IL-18 response. It remains to be determined whether Vpu interacts with the channel protein directly causing the channel to open or whether the effect on Kv activity is indirect.

On the other hand, our results provide evidence that HIV-1 gp41 activates the NAIP/NLRC4 inflammasome and triggers mainly IL-18 production. HIV gp41 does not trigger a “priming signal” but rather uses a “one-step” inflammasome activation by directly interacting intracellularly with NAIP/NLRC4. HIV gp41 is similar to type III secretion rod and needle proteins, in that it disrupts plasma membrane integrity and regulation and is recognized by NAIP via its conserved hydrophobic cytoplasmic domain. This conserved mechanism promotes NAIP interaction with NLRC4 and formation of an oligomeric NLRC4 inflammasome complex, resulting in activation of caspase-1 and IL-18 secretion. This is the first study showing that human NAIP can recognize viral proteins. Thus, we can conclude that NAIP likely acts as a broad-spectrum innate immune surveillance system that detects microbial proteins from bacteria and viruses which disrupt membrane stability. Given that diverse viruses likely utilize conserved mechanisms for directing viroporin incorporation into the cell and the ability of RNA viruses such as influenza virus and HIV to utilise lipid rafts for assembly, and cell entry there could be a much broader range of NAIP detection than previously thought [66–68].

Interestingly, localisation of gp41 to lipid rafts seems to be required for NAIP activation. This is unusual as most NAIP ligands are soluble, but its association with non-soluble ligands has previously been reported. In particular one of tis ligands in Shiga-toxigenic *E*.*coli* 0113:H21 strain, flagellin (which is a NAIP ligand) has been shown to mediate invasion which is lipid-raft-dependent [69]. On the other hand, topology of HIV gp41 C-terminus, which we have identified as the ligand for NAIP, is proposed to be either completely intracytoplasmic or with an extracellular position. Therefore, it could be that there is a transient exposure of gp41 C-terminus in lipid rafts during membrane fusion. Its concentration within lipid rafts could cause lipid dysregulation and internalization, subsequently interacting with NAIP (Fig 6C); what is even more interesting is that gp41 has been previously shown to directly interact with other host proteins intracellularly, in particular TAK1 [70].

Our results highlight the divergent production of IL-1β and IL-18 by Vpu and gp41 from HIV-1, which could account for different inflammatory manifestations during HIV infection. This discordant/staggered activation of inflammasome cytokines could be crucial for the triggering of chronic HIV-1 inflammation and immune activation in the development of non-AIDS conditions such as cardiovascular, respiratory and neurologic diseases which leads to increased mortality in HIV-1 positive patients [71,72]. The early, IL-1β production in response to Vpu could be beneficial for the clearance of the virus, whereas the delayed IL-18 response via Vpu and gp41 could have detrimental effects for CD4+ T-cells. Since IL-18 has been shown to increase CXCR4 expression as well as the apoptosis TRAIL in PBMCs of HIV patients, it would suggest that raised IL-18 must promote viral replication (thus impaired response to ART) and apoptosis and subsequent depletion of CD4+ T-cells leading to chronic inflammation and immune activation. Therefore, virological treatment failure and persistent IL-18 raised levels could be due to discordant innate immune recognition of gp41, lending support to possibly targeting IL-18 in HIV patients [73]. Our novel mechanistic insight sheds light on the mechanisms by which HIV-1 produces these cytokines, defining targets that could be relevant in the fight against HIV-1 mediated pathology.

## Methods

### Virus production and infectivity assay

HIV_NL4-3_ (X4-tropic) and HIV-1 _R8Bal_ (R5-tropic) were prepared by transfection of 1×10^6^ 293T cells per well in a 24-well plate with 1 μg of the appropriate full-length infectious HIV plasmid using the FuGene6 transfection reagent (Roche). Virus-containing supernatants were passed through a through 0.45 μm pore-size nitrocellulose membrane (Spin-X; Corning) filter to remove cellular debris and precipitated in polyethylene glycol at 4°C. Precipitated virus was then centrifuged at 14,000 g for 20 minutes, re-suspended in PBS, and frozen at −80°C until use.

HIV-1 pseudotyped viruses were generated as follows: 293T cells were transfected with 100 ng of pNL4-3 ΔVpu (HIV ΔVpu) (NIH AIDS Reagents Program, NIBSC, UK) or pNLCH Δenv (HIV Δenv) (ViiV Healthcare) or pNLCH-wtHIV (ViiV Healthcare) in the presence of the pVSV-G envelope vector (vesicular stomatitis virus glycoprotein G), using GeneJuice (MercK Millipore., USA). After 48 h, the supernatant was harvested, filtered through 0.22-μm filters, collected and frozen at −80°C until use.

The level of p24 protein in cell culture supernatants of HIV virus was determined by p24-specific ELISA assay, performed by HIV-1 p24^CA^ Antigen Capture Assay kit from AIDS & Cancer Virus Program, which measures the amount of viral capsid protein in the supernatant. TCID50 of the viral stocks to determine the actual MOI was also performed.

Human macrophages (1 x 10^6^ per well) when infected with virus were incubated with (50ng of p24, MOI = 1). For pharmacological inhibition studies cells were treated with inhibitors for 1hr before HIV-1 infection and then cultured in the presence of drugs.

### Cell culture

Human peripheral blood mononuclear cells (PBMCs) were isolated from buffy coat by Ficoll-Paque Plus. Primary human monocytes were isolated from PBMC using the Miltenyi Biotec MACS magnetic cell separation system in order to isolate the CD14+ fraction From the PBMCs or via negative selection using the easystep human monocyte enrichment kit (Stemcell technologies). Monocytes were maintained in RPMI medium containing 10% FBS, and 1% nonessential amino acids. Primary human macrophages were differentiated from monocytes by culturing in medium containing 100ng/ml recombinant M-CSF (R&D systems) for 5 days. Our experiments showed no difference in macrophages coming either from positive or negative selection.

The human biological samples were sourced ethically, and their research use was in accord with the terms of the informed consents.

### Drug optimization/Viability assays

Human macrophages were treated with drugs, EIPA, benzamil, and verapamil, MgTX, Sotalol (at various concentrations for 12hr or 24hr). Drug-treated cells were then incubated with 0.2% Trypan blue for 1min. Cells were washed three times with PBS, mounted onto glass slides and viewed immediately on a Nikon microscope.

### RNA interference

Primary monocyte differentiated macrophages (MDMs) were electroporated (nucleofection) similar to a study by Gui et. al. [74] using the Human *Macrophage Nucleofector* Kit from Lonza and the 4D Nucleofection core unit with 30pmol of control siRNA, or siRNA either for TLR2 (NM_003264–00074935 and NM_003264–00074937), TLR3 (NM_003265–00231804 and NM_003265–00231806), TLR4 (NM_138554–00122250 and NM_138554–00122252), TLR8 (NM_016610–00064920 and NM_016610–00064925), NLRC4 (NM_021209/00184452 and NM_021209/00184453), NLRP1 (NM_001033053/00151115 and NM_001033053/00151117) NLRP3 (NM_001127461/00329604 and NM_001127461/00329605), NLRC5 (NM_032206/00359503 and NM_032206/00191925) or NAIP (NM_004536/0056857 and NM_004536/0056860). After 48 h the level of silencing was determined by western blotting. Electroporation of human macrophages revealed high efficiency (40%) combined with low mortality. The cells were then sorted by flow cytometry, evaluated with the Cellquest software (BD Biosciences, San Jose, CA) to determine the proportion of fluorescent cells as described in [75]. In order to achieve the same knockdown effect in all species of siRNAs, the experiments were performed several times (in some cases we had to repeat 8 times to achieve the same level of knock down as the other NLRPs. All siRNAs used were Mission siRNAs from Sigma.

### Fluorescence resonance energy transfer

FRET is a non-invasive imaging technique used to determine molecular proximity. FRET can occur over 1–10 nm distances, and effectively increases the resolution of light microscopy to the molecular level. FRET was measured in terms of dequenching of donor fluorescence after complete photo-bleaching of the acceptor fluorophore. Increased donor fluorescence after complete destruction of the acceptor indicates association between the two molecules of interest.

### Confocal microscopy

Monocytes or differentiated macrophages were nucleofected with 2 μg of pVpu, at 24 h post transfection cells were rinsed twice in PBS/0.02% BSA, prior to fixation with 4% formaldehyde for 15 min. The cells were fixed in order to prevent potential re-organisation of the `proteins during the course of the experiment. Cells were permeabilised using PBS/0.02% BSA/0.02% Saponin and labelled with antibodies for NLRC4, NLRP3 directly labelled with the appropriate fluorophore. We have validated our confocal experiments with isotype control antibodies as well as with only secondary antibody. Furthermore NLRP3, NAIP and NLRC4 antibodies have been validated for non-specific binding by using NLRP3, NAIP and NLRC4 knockdown cells respectively (S2 Fig). The results showed no nonspecific binding.

Cells were imaged on a Carl Zeiss, Inc. LSM510 META confocal microscope (with an Axiovert 200 fluorescent microscope) using a 1.4 NA 63x Zeiss objective. The images were analysed using LSM 2.5 image analysis software (Carl Zeiss, Inc.).

In order to quantify the degree of co-localization, we used ImageJ software (MacBiophotonics). The analysis uses Costes’ approach. This allows for the calculation of Pearson’s correlation coefficient *R*(obs), which also accounts for any random overlay of pixels by generating the mean correlation coefficient *R*(rand) between *n* images that have identical average pixel intensity to the original images, but a random distribution of pixels. Costes’ randomisation method calculates the statistical significance of the Pearson’s correlation coefficient. It returns a significance (p-value) expressed as a percentage.

### Cytokine assays

Cell supernatants were collected after each stimulation and frozen until the assays were performed. The IL-18, human IL-1β ELISA (Human IL-1-beta/IL-1F2 DuoSet ELISA) and pro-IL-1β kits (R&D systems) were used.

### Co-immunoprecipitation assays

Macrophage cells lysates were prepared by lysing cells with buffer (50 mM Tris-HCl, pH 7.5, 50 mM Nacl, 0.1% NonidetP40, 1 mM MgCl_2,_ 1mM CaCl_2_ and 2mM PMSF). Lysates were immunoprecipitated with anti gp41, or control mouse immunoglobulin G (IgG) (Invitrogen) or anti-Flag (anti Myc-DDK tag antibody) (Origene, USA) with Protein-A Sepharose (GE Healthcare, Milwaukee, WI, USA).

### Chemicals/antibodies/reagents

All fine chemicals were obtained from Sigma-Aldrich. Anti-caspase 1 p20 rabbit polyclonal antibody, anti-NLRP3 rabbit polyclonal antibody (H-66), anti-NLRP3, goat polyclonal antibody (M-12) were purchased from Santa Cruz Biotechnology (CA, USA). Anti-Human NAIP (CL541609) was from Thermofisher Scientific Ltd (USA). NAIP (ab25968) was from abcam, anti-NLRC4 rabbit polyclonal **(**06–1125*)* was from Merck. Anti-NLRC4 mAb (F-3) was purchased from Santa Cruz. Anti-GM130 mAb was from BD Biosciences, Anti-GM130 rabbit polyclonal antibody (G7295) was from Sigma Aldrich. Anti-GM130 goat antibody conjugated to Alexa 546 was from Abcam. Anti-Kv1.3 (G-9) mAb was from Santa Cruz, anti Kv1.3(GTX16637) rabbit polyclonal IgG was from GeneTex. Anti-IL1β antibody (NBP1-42767) was obtained from Novus (USA). Rabbit polyclonal to HIV1 gp41 (ab30755) and anti-Vpu antibody (ab81532) was obtained from Abcam. HIV-1 gp41, HIV-1 gp120 as well as HIV-1 anti-gp41 mAb (7H6) were obtained from the NIH AIDS Reagents Program, NIBSC, UK. HIV-1 Vpu was obtained from Biosource. Expression clones for HIV-1 gp41 (pgp41), gp120 (pg120) and Vpu (pVpu) with a C-terminal myc-DDK tag were obtained from Origene, U.S.A.

Macrophages were either stimulated with 50ng/ml of recombinant HIV-1 gp41, Vpu complexed with lyovec from Invivogen (for internalisation) or transfected with 2 μg expression clones pgp41and pVpu, with similar results.

The concentration of the recombinant proteins used were in accordance with a study by Santosuosso et al. which had shown high levels of another envelope protein gp120 in patients with chronic HIV infection^52^ (up to 2200 pg/ml gp120 in the peripheral blood of untreated patients and 218ng/ml in lymph nodes of untreated animals).

Furthermore, a dose response of different concentrations of gp41 and Vpu was used to trigger IL18 and IL1β secretion as well as determine cell toxicity using trypan blue prior to the concentration selected for stimulation (S3 **Fig**).

In all the reagents used no endotoxin could be detected, using the Limulus amebocyte lysate (LAL) assay.

### Super-resolution microscopy

Super-resolution dSTORM were performed on a Zeiss ELYRA P.1 system (Carl Zeiss, USA). Images were acquired with a Plan-Apochromat 633/1.40 oil immersion objective and an Andor iXon 885 EMCCD camera. Fifteen images per plane (five phases, three rotations) and 0.125 mm z section of 3 mm height were required for generating super resolution images. Raw images were reconstructed and processed to demonstrate structure with greater resolution by the ZEN 2011 microscope software (Carl Zeiss,Germany). For cluster analysis, Ripley’s L function was determined using ImageJ.

### Protein purification/expression purification of recombinant peptides

The NAIP protein was purified from human cell lysate using an NHS activated column conjugated to a NAIP antibody (LS-B455) in order to pull out NAIP from the cell lysate. The elution buffer used was 100 mM triethanolamine pH 10. The pH of the eluted protein was adjusted to pH 7 and was buffer exchanged via dialysis for 6hr at 4C with a buffer containing 50mM Hepes,150mM NaCl, 5% Glycerol, 1mM DTT, 0.02mM ZnCl_2._

In order to determine suitable buffer conditions, prior to buffer exchange a Protein Stability screen from Molecular Dimensions (Newmarket, UK) had been used to provide information on the effect of various environmental factors that will have on the denaturation or aggregation of NAIP protein. The Durham pH Screen which deconvolutes buffer molecule (28 different buffers) and pH effects pH (4–11) as well as the Durham Salt Screen (30 different salts), including a range of lanathanides were included in the screen in order to determine the buffer used for dialysis. A small amount of aggregated protein formed was removed by gel filtration. Prior to use protein aggregation was determined by a fast high throughput plate based fluorometric assay from Abcam. NAIP was buffer exchanged kept at 4°C and was used within 24hr.

### HIV gp41 recombinant peptides

gp41 FP, gp41 ectodomain, gp41(APR680), gp41MPER, gp41 (APR7022) were from the AIDS Research Reagent Program NIAID, gp41CHR, gp41 P5181, gp41 P5079, gp41 TM/CT, gp41V113 were custom made from Anaspec. The gp41 constructs were non glycosylated and created analogously, by cloning the respective gp41CD sequences into vector pGEV. Sequence fidelity of constructs was verified by DNA sequencing. The recombinant plasmids were expressed in E.coli and then purified.

### Surface plasmon resonance (SPR) analysis

The interactions between gp41 and NAIP were analysed with a BIAcore 3000 (Uppsala, Sweden). The BIAcore system is equipped with the sensory chip CM5, a small metal chip with a carboxymethyldextran surface, to allow ligand immobilization via native NH2. An amine coupling kit containing N-hydroxysuccinimide (NHS), N-ethyl-N9-[(3-dimethylamino)-propyl]-carbodiimide hydrochloride (EDC), and ethanolamine-HCl (Amersham Pharmacia Biotech) was used to immobilize the ligand to the chip. The NAIP was immobilized on the sensory chip CM5 to measure its interaction with different regions of gp41. The analyses of the kinetic parameters were performed by using BIA EVALUATION 3.0, software designed to analyze experimental sensor graph data for kinetics and affinity of interactions, according to the manufacturer’s manual.

## Supporting information

S1 Fig A dose response using different concentrations of Vpu or gp41. A dose response using different concentrations of Vpu (A) or gp41 (LyoVec was used as a vehicle) (B) were tested on MDMs for IL-1β and IL-18 secretion using human IL-18 and human IL-1β ELISA kits (eBiosciences) (A vehicle control was also performed (C). MDMs either infected with HIV-1 for 12 hr or stimulated with 100 ng/ml Vpu or gp41 for 12 hr were lysed and cell lysate was analysed for Vpu or gp41 protein levels using western blotting, followed by quantification using Image Studio Lite (Licor) and normalized to internal control (β-actin) from 3 western blots (D). Supernatants from MDMs either infected with HIV-1 for 12 hr or stimulated with 50 ng/ml Vpu or gp41 were assessed for IL-1β and IL-18 secretion using ELISA (E). The data presented is the mean of three independent experiments ± SD ** *p* < 0.005 and ***, *p* < 0.001 indicate statistically significant differences.
(TIF)

S2 Fig IL-1β and IL-18 secretion in response to HIV-1 Vpu and gp41. Monocyte-derived macrophages (MDMs) (1 x 10^6^) were stimulated with HIV-1 Vpu (50 ng/ml) (A) or HIV gp41 (50 ng/ml) (B) for 48 hrs. Supernatants were collected at 4, 8, 12, 24, 36 and 48 hrs and analysed for IL-1β and IL-18 using ELISA. The data presented is the mean of three independent experiments. MDMs were pre-treated for 1 hr with NLRP3 inflammasome inhibitor MCC950 (0.01 μM) or Ac-YVAD-cmk caspase-1 inhibitor (10 μg/ml) and then stimulated with Vpu or gp41 for 12 hr (C) or 24 hr (D). Supernatants were collected and tested for IL-1β and IL-18 secretion (C,D). HIVwt VSV-G and HIV 1 VSV-G pseudotyped mutants lacking Env expression. (HIV1Δenv) or lacking Vpu (HIV-1 ΔVpu) were used to infect MDMs for 12 hr. Supernatant was collected and analysed for IL-1β (E) and IL-18 (F) using ELISA. The data represent the mean of three independent experiments ± SD (*n* = 3 sets of macrophages) yielding consistent results. **, *p* < 0.005 and ***, *p* < 0.001 indicate statistically significant differences.
(TIF)

S3 Fig A dose response using different concentrations of ion channel inhibitors. Monocyte-derived macrophages (MDMs) (1 x 10^6^) were stimulated with HIV-1 Vpu (50 ng/ml) and different concentrations of ion channel inhibitors, such as EIPA (A), Benzamil (B), TEA (C), BAPTA (D), Amantadine (E), Verapamil (F), Solatol (G), Ba2+(H), 4AP(I), MgTX(J), or GSK’816A (K).
(TIF)

S4 Fig Effect of traditional inflammasome “Signal 2” inhibitors on HIV-induced IL-1β & IL-18 processing. Monocyte-derived macrophages (MDMs) (1 x 10^6^) were infected with HIV-1 for 12 h in the presence or absence of Cathepsin B inhibitor (CA-074) (100 μM), DPI (20μM), NAC (20mM) chloropromazine (50μg/ml) and the presence of pro-IL-1β, pro-IL-18, cleaved IL-1β and cleaved IL-18 was investigated via western blotting.
(TIF)
