# Supplementary figures and images for: Differential recognition of HIV-stimulated IL-1β and IL-18 secretion through NLR and NAIP signalling in monocyte-derived macrophages
Source: PLoS Pathog. 2021 Apr 16;17(4):e1009417. doi: 10.1371/journal.ppat.1009417 (PMC8109768; doi:10.1371/journal.ppat.1009417)

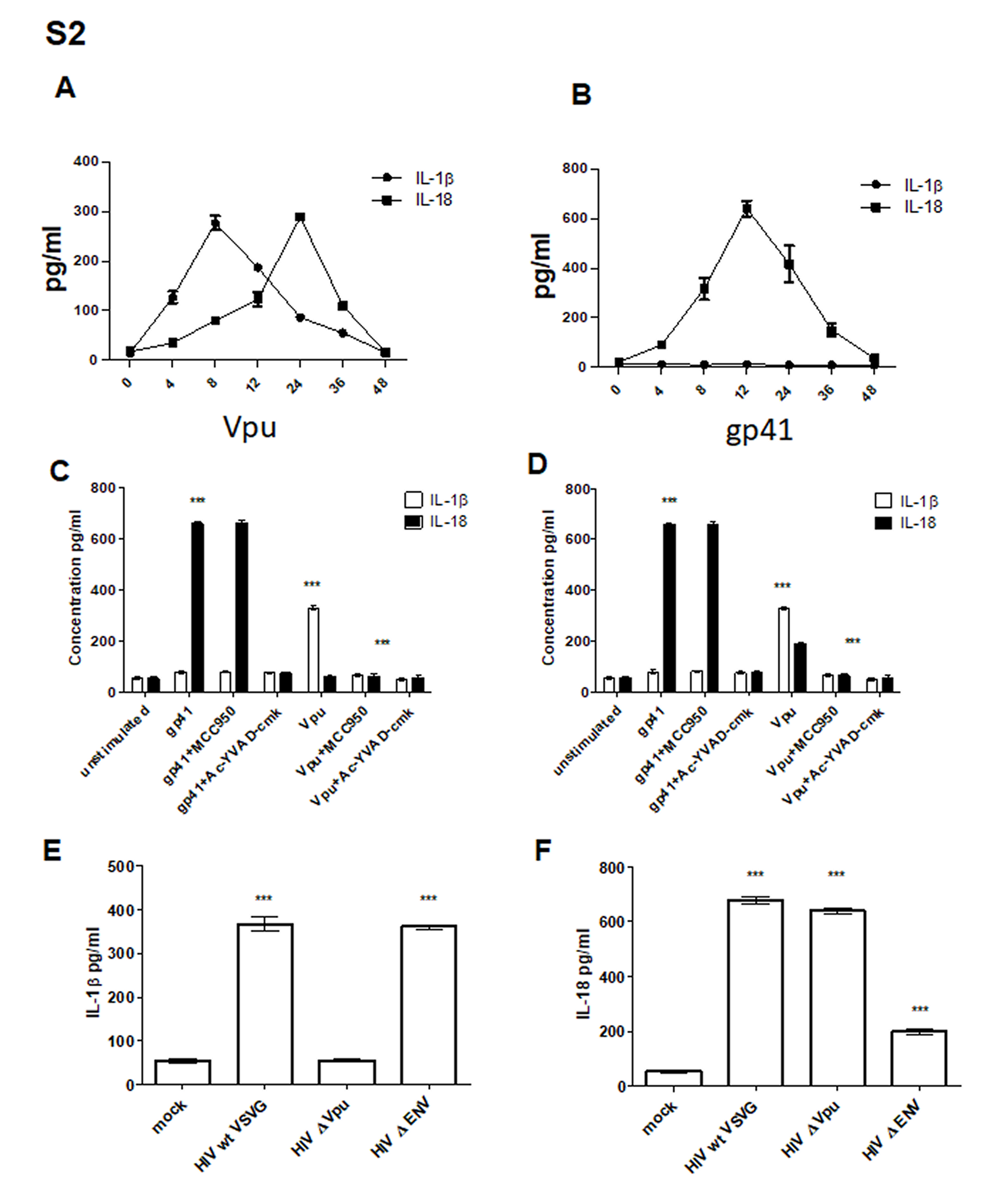

Supplement: S2 Fig — Monocyte-derived macrophages (MDMs) (1 x 10^6^) were stimulated with HIV-1 Vpu (50 ng/ml) (A) or HIV gp41 (50 ng/ml) (B) for 48 hrs. Supernatants were collected at 4, 8, 12, 24, 36 and 48 hrs and analysed for IL-1β and IL-18 using ELISA. The data presented is the mean of three independent experiments. MDMs were pre-treated for 1 hr with NLRP3 inflammasome inhibitor MCC950 (0.01 μM) or Ac-YVAD-cmk caspase-1 inhibitor (10 μg/ml) and then stimulated with Vpu or gp41 for 12 hr (C) or 24 hr (D). Supernatants were collected and tested for IL-1β and IL-18 secretion (C,D). HIVwt VSV-G and HIV 1 VSV-G pseudotyped mutants lacking Env expression. (HIV1Δenv) or lacking Vpu (HIV-1 ΔVpu) were used to infect MDMs for 12 hr. Supernatant was collected and analysed for IL-1β (E) and IL-18 (F) using ELISA. The data represent the mean of three independent experiments ± SD (n = 3 sets of macrophages) yielding consistent results. **, p < 0.005 and ***, p < 0.001 indicate statistically significant differences. (TIF) [file ppat.1009417.s002.tif]

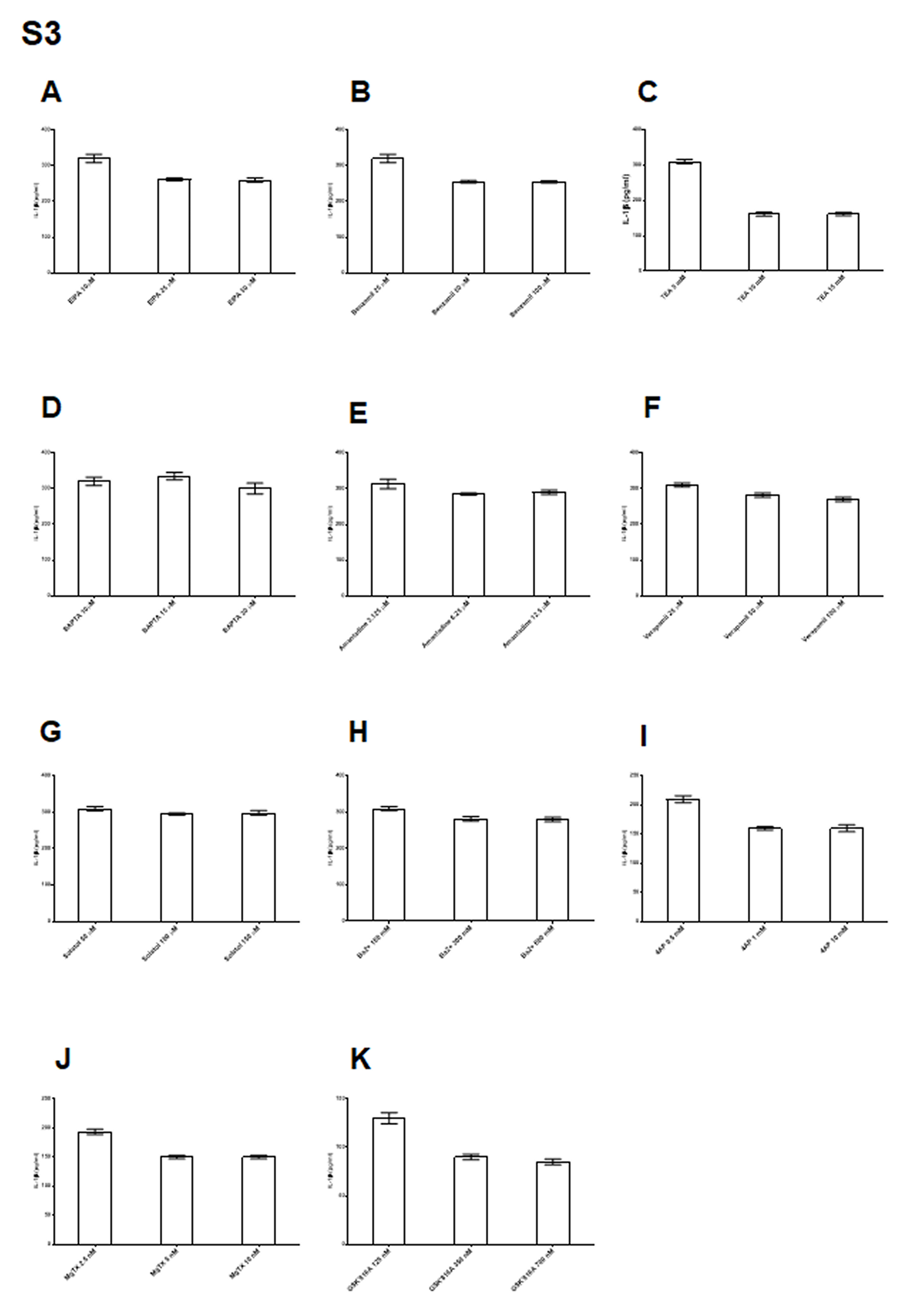

Supplement: S3 Fig — Monocyte-derived macrophages (MDMs) (1 x 10^6^) were stimulated with HIV-1 Vpu (50 ng/ml) and different concentrations of ion channel inhibitors, such as EIPA (A), Benzamil (B), TEA (C), BAPTA (D), Amantadine (E), Verapamil (F), Solatol (G), Ba2+(H), 4AP(I), MgTX(J), or GSK’816A (K). (TIF) [file ppat.1009417.s003.tif]

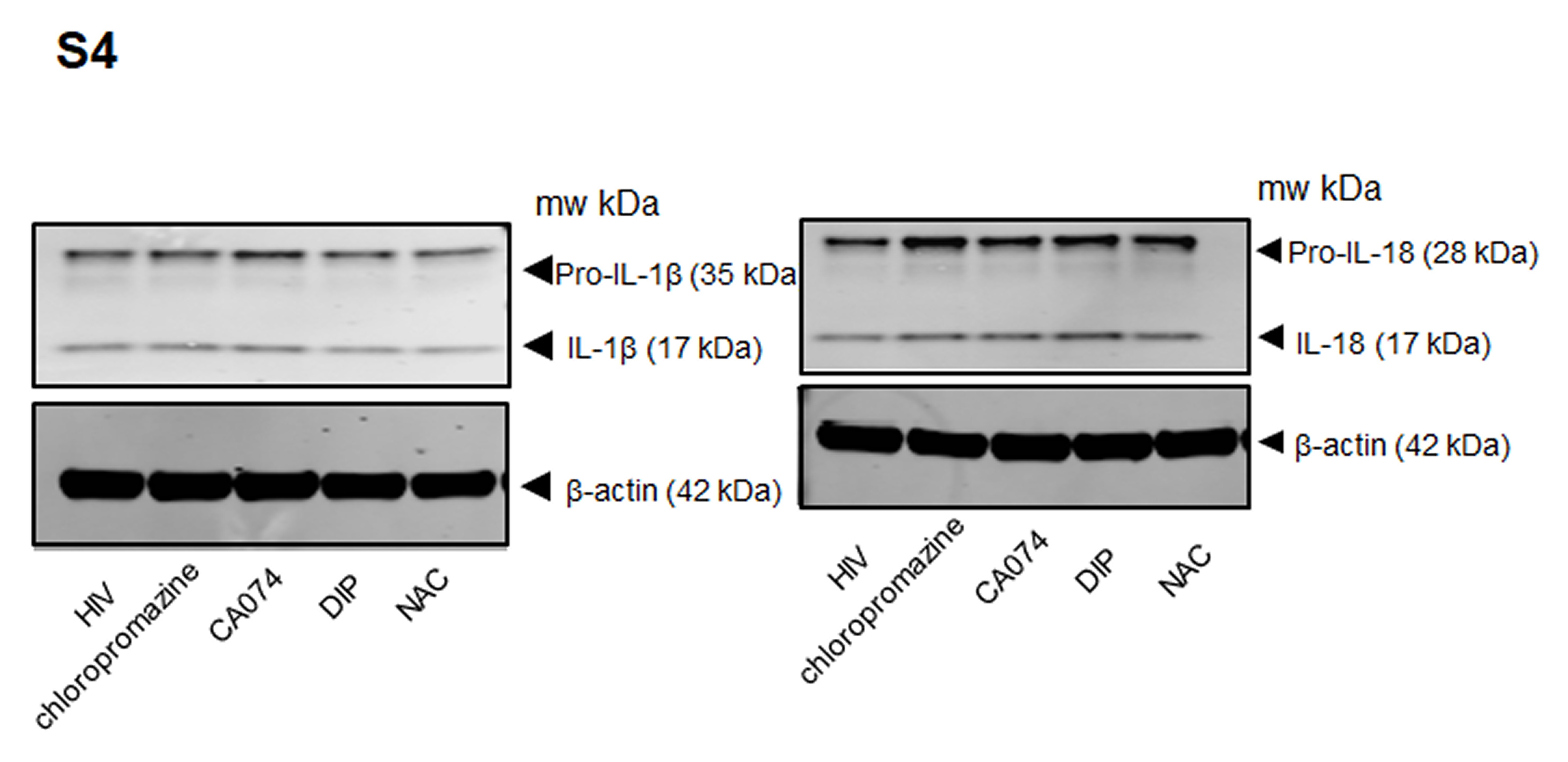

Supplement: S4 Fig — Monocyte-derived macrophages (MDMs) (1 x 10^6^) were infected with HIV-1 for 12 h in the presence or absence of Cathepsin B inhibitor (CA-074) (100 μM), DPI (20μM), NAC (20mM) chloropromazine (50μg/ml) and the presence of pro-IL-1β, pro-IL-18, cleaved IL-1β and cleaved IL-18 was investigated via western blotting. (TIF) [file ppat.1009417.s004.tif]
